# Forgetting fear associations through tES: which memory process might be critical?

**DOI:** 10.1038/tp.2017.26

**Published:** 2017-02-28

**Authors:** C M Vicario, M A Nitsche, K Felmingham

**Affiliations:** 1 School of Psychology, University of Tasmania, Hobart, TAS, Australia; 2 Wolfson Centre for Clinical and Cognitive Neuroscience, School of Psychology, Bangor University, Bangor, UK; 3 Leibniz Research Centre for Working Environment and Human Factors, Dortmund, Germany; 4 University Medical Hospital Bergmannsheil, Bochum, Germany

We read with interest the article by Abend and colleagues^1^ titled ‘Modulation of fear extinction processes using transcranial electrical stimulation’, which adds relevant insights to the extremely limited literature on the effect of non-invasive brain stimulation on fear extinction mechanisms. Given the interest in using brain stimulation for treatment of anxiety disorders and post-traumatic stress disorder, research examining the impact of non-invasive brain stimulation on mechanisms involved in the maintenance and treatment of these disorders is critical.

In their study, Abend and colleagues examined the effects of two different types of non-invasive transcranial electrical stimulation (tES; that is, direct current (DC) and alternating current (AC) stimulation) on fear extinction processes in healthy humans to assess a potential relevance/application of these protocols in therapeutic context. Thus, the authors decided to test three separate groups of participants (that is, DC, AC and Sham stimulation) by targeting the medial prefrontal cortex, which is known to mediate fear extinction mechanisms (for example, Milad and Quirk^2^), in a 3-day protocol. On the basis of previous research, they expected that excitability-enhancing anodal tDCS over the ventromedial prefrontal cortex would improve extinction, most probably via activation of GABAergic neurons of the amygdala,^3^ and that low-frequency tACS during extinction might reduce fear memory consolidation via long-term depression-like effects. Skin conductance and self-report responses were examined to assess the effect of the provided treatments in fear extinction.

The results are not in line with the predicted outcomes as AC stimulation enhanced the expression of fear responses following extinction, whereas DC stimulation led to overgeneralization of the fear response to non-reinforced stimuli for the skin conductance parameter, compared to the sham stimulation.

The authors provide different, not mutually exclusive, explanations for interpreting the reported results (for example, the temporal momentum associated to the tES delivery and/or the involvement of other brain regions than the mesial prefrontal cortex and/or brain circuits). All these aspects make good sense, given the fact that the dorsal anterior cingulate cortex, which is situated nearby the mesial prefrontal cortex, has antagonistic effects on extinction,^4^ and that extinction might be timing-specific. These aspects call for spatially and temporally more specific stimulation protocols.

The application of tES for 20 min, during the fear extinction-learning phase, as it was done in that study, raises more issues that might add further insights to the current discussion on these unexpected results. The process of fear association learning, as well as any learning process, consists of two distinct mechanisms, namely encoding and consolidation. According to definitions of eminent scholars in the field, the encoding process refers to ‘operations that take place when the individual originally experiences an event initiate the storage of information representing the event in episodic memory’^5^ the consolidation process refers to ‘the idea that neural processes transpiring after the initial registration of information contribute to the permanent storage of memory’.^6^

The research design proposed by Abend and colleagues does not allow to discern whether the reported results (i) are due to a modulation of the *encoding* process, which takes place during the fear extinction phase; (ii) reflect a tES after-effect phenomenon, that might last for some hours (for example, refs 7, 8, 9), and therefore reflect the influence of tES on the *consolidation* process; (iii) reflect the influence of tES on both processes (see Figure 1 for a general schema).

The application of tES in a research design that separates the ‘*encoding*’ from the ‘*consolidation*’ process might provide important insights for the current discussion, as distinct mechanisms (for example, Cho *et al.*^10^) for these two processes have been described with regard to extinction of fear memories. At the same time, it would provide highly relevant insights for the application of tES in the clinical field, as it might clarify which process should be targeted by electrical stimulation to empower its effect of a memory extinction therapy.

## Figures and Tables

**Figure 1:**
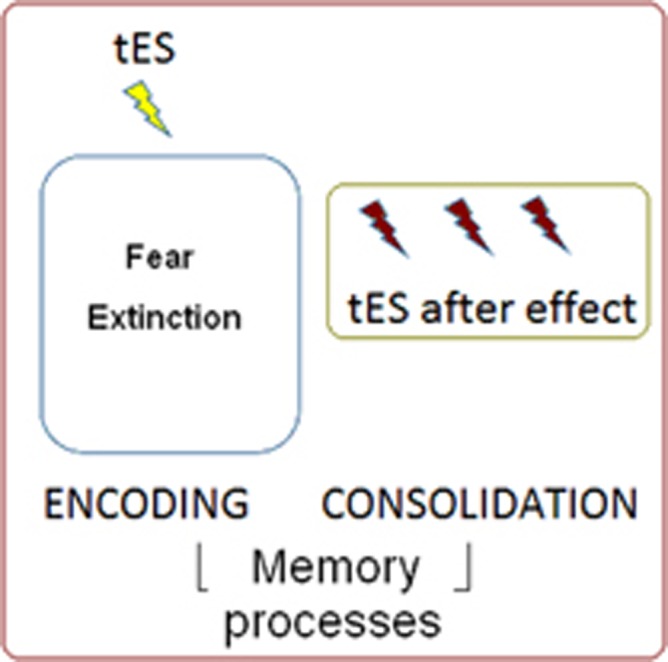
Diagram of transcranial electrical stimulation (tES) effects on memory processes (that is, encoding and consolidation) during and after the extinction phase.
